# Crystal structure of the Ilheus virus helicase: implications for enzyme function and drug design

**DOI:** 10.1186/s13578-022-00777-8

**Published:** 2022-04-15

**Authors:** De-Ping Wang, Mei-Yue Wang, Yong-Mei Li, Wen Shu, Wen Cui, Fang-Ying Jiang, Xin Zhou, Wen-Ming Wang, Ji-Min Cao

**Affiliations:** 1 Key Laboratory of Cellular Physiology at Shanxi Medical University, Ministry of Education, and the Department of Physiology, Shanxi Medical University, Taiyuan, China; 2 Institute of Life Sciences, Chongqing Medical University, Chongqing, China; 3 Institute of Molecular Science, Shanxi University, Taiyuan, China

**Keywords:** Ilheus virus, NS3 helicase, Crystal structure, ATP hydrolysis, Molecular docking

## Abstract

**Background:**

The Ilheus virus (ILHV) is an encephalitis associated arthropod-borne flavivirus. It was first identified in Ilheus City in the northeast Brazil before spreading to a wider geographic range. No specific vaccines or drugs are currently available for the treatment of ILHV infections. The ILHV helicase, like other flavivirus helicases, possesses 5ʹ-triphosphatase activity. This allows it to perform ATP hydrolysis to generate energy as well as sustain double-stranded RNA’s unwinding during ILHV genome replication. Thus, ILHV helicase is an ideal target for inhibitor design.

**Results:**

We determined the crystal structure of the ILHV helicase at 1.75-Å resolution. We then conducted molecular docking of ATP-Mn^2+^ to the ILHV helicase. Comparisons with related flavivirus helicases indicated that both the NTP and the RNA-ILHV helicase binding sites were conserved across intra-genus species. This suggested that ILHV helicase adopts an identical mode in recognizing ATP/Mn^2+^. However, the P-loop in the active site showed a distinctive conformation; reflecting a different local structural rearrangement. ILHV helicase enzymatic activity was also characterized. This was found to be relatively lower than that of the DENV, ZIKV, MVE, and ALSV helicases. Our structure-guided mutagenesis revealed that R26A, E110A, and Q280A greatly reduced the ATPase activities. Moreover, we docked two small molecule inhibitors of DENV helicase (ST-610 and suramin) to the ILHV helicase and found that these two molecules had the potential to inhibit the activity of ILHV helicase as well.

**Conclusion:**

High-resolution ILHV helicase structural analysis demonstrates the key amino acids of ATPase activities and could be useful for the design of inhibitors targeting the helicase of ILHV.

**Supplementary Information:**

The online version contains supplementary material available at 10.1186/s13578-022-00777-8.

## Background

Many flaviviruses caused significant infections and diseases in humans among the emerging diseases. Dengue virus (DENV), Yellow fever virus (YFV), and Zika virus (ZIKV) belong to the flavivirus, which have been responsible for massive outbreaks and attracted public attention [1–4]. Certain strains of public health threatening flaviviruses have been isolated in Brazil. These Brazilian flavivirus strains, all found to cause sporadic cases of human infection, specifically include Cacicapore virus (CPCV), Bussuquara virus (BUSV), Rocio virus (ROCV), Saint Louis encephalitis virus (SLEV), and Ilheus virus (ILHV) [5–7]. ILHV was first identified in the northeast of Brazil in 1994 near the Ilheus City. It was discovered in Aedes and *Psorophora* spp. Mosquitoes. ILHV has been transmitted over a wide geographic range encompassing the Caribbean, Central America, and South America; particularly in Brazil and Trinidad [8, 9]. Symptoms of ILHV infection include fever, headache, myalgia, arthralgia, and photophobia. ILHV’s impact upon the central nervous system (CNS) involvement has also been reported in a handful of documented human cases [8, 10, 11]. This ties to encephalitic diagnoses that can lead to fatal outcomes [8]. Thus, although the current number of ILHV infection caused is low, an increasing number of arbovirus infected patients show unusual clinical manifestations [12–17]. Notably, arboviruses have the potential to invade the CNS, resulting in long-term neurological sequelae. As a result, their potential danger to human health cannot be ignored [18, 19]. Therefore, in-depth studies are thus warranted on the proteins of these dangerous pathogens.

ILHV is a single-stranded and positive-sense RNA virus. Its seven nonstructural proteins (NS1, NS2a, NS2b, NS3, NS4a, NS4b, and NS5) are critical for virus replication, virion assembly, and host invasion. The flavivirus NS3 protein is one of the most important nonstructural proteins because of its multiple enzymatic activities. It contains an N-terminal protease domain and a C-terminal RNA helicase/NTPase domain. The RNA helicase/NTPase domain in the C-terminal plays a critical role in RNA synthesis and replication of the viral genome. NS3 helicase activity is essential for the RNA unwinding and is responsible for hydrolyzing the triphosphate at the 5‏‏‏′ end of RNA [9, 20–22]. Many studies are underway on helicase inhibitors. To date, a range of flavivirus NS3 helicase structures have been reported, including those of Zika virus, hepatitis C virus, yellow fever virus, West Nile virus, and Dengue virus [9, 20–24]. Currently, no effective or special treatment is available for ILHV infections.

In the present study, we solved the three-dimensional structure of ILHV helicase, which provides an accurate model for rational drug design against IHLV infection. Moreover, we compared the structure of ILHV helicase with that of other flaviviruses and analyzed the similarities and differences. The ILHV helicase and ATP complex was constructed based on the molecular docking. An ATPase activity assay of the wild type helicase and mutant helicase (R26A, E110A, Q280A) was performed to identify critical residues for ILHV helicase ATPase activity. In addition, we also docked two small molecule inhibitors of DENV helicase named ST-610 and suramin to the ILHV helicase and found that they may have the potential to inhibit the ILHV helicase activity as well. These studies may not only help elucidate how ILHV helicase recognizes its substrates during replication, but also provide structural insights for screening antiviral compounds.

## Results

### Overall structure of ILHV helicase

The ILHV helicase crystals belong to the *P4*_*1*_*2*_*1*_*2* space group and the diffraction data were collected at 1.75 Å resolution. Data collection and refinement statistics are listed in Additional file 1: Table S1. Notably, the crystal structure of ILHV helicase had one molecule in an asymmetric unit and no dimer or oligomer formation was identified in the crystals through crystallographic packing; this result was consistent with the size exclusion chromatography results (Additional file 1: Fig. S1), indicating that ILHV helicase functions as a monomer.

**Fig. 1:**
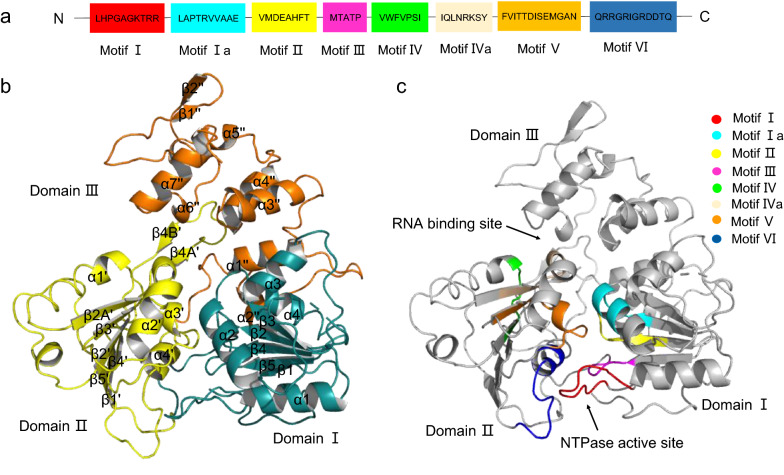
**a** Diagram of the eight conserved motifs of ILHV helicase. **b** Ribbon model of the crystal structure of ILHV helicase with annotated secondary-structure elements. **c** Ribbon model of the ILHV helicase structure with the conserved motifs highlighted using the indicated color codes

The crystal structure of ILHV helicase was in a flattened triangular shape and contained three domains: domain I and II in the N-terminal and domain III in the C-terminal (Fig. 1a–c). Domains I and II were RecA-like domains with an α/β fold. Domain I was composed of six parallel β-sheets (β1, β2, β3, β4, β5, and β2A), which were stacked between a large number of loops and four α-helices (α1, α2, α3, and α4). β2A was not showed because the residues were not visible in the electron density maps (Fig. 1b). Domain II was composed of six parallel β-sheets (β1ʹ, β2ʹ, β3ʹ, β4ʹ, β5ʹ, and β2Aʹ) and sandwiched by four α-helices (α1ʹ, α2ʹ, α3ʹ, and α4ʹ). A β-hairpin composed of a pair of antiparallel β-sheets protrudes from domain II and interacted with domain III (β4Aʹ, and β4Bʹ). Domain III mainly consisted of a seven-α-helix bundle (α1ʺ, α2ʺ, α3ʺ, α4ʺ, α5ʺ, α6ʺ, and α7ʺ). A pair of antiparallel β strands (β1ʺ and β2ʺ) in Domain III was partially exposed to the solvent, broadening this domain (Fig. 1b). As evidenced in their clear inter-domain clefts, these three domains were well distinguished.

There were eight highly conserved structural motifs in the ILHV helicase: motifs I, Ia, II, III, IV, IVa, V, and VI (Fig. 1a, c). All conserved SF2 helicase motifs lay in the cleft formed by domains I and II. Domain I contained motifs I (P-loop/ Walker A), Ia, II (Walker B), and III. Domain II contained motifs IV, IVa, V, and VI. The binding site for ATP was located at the interface between Domains I and II, the cleft between Domain II and Domain III was the RNA binding site (Fig. 1c).

### Comparison of ILHV helicase with other flavivirus helicases

**Fig. 2:**
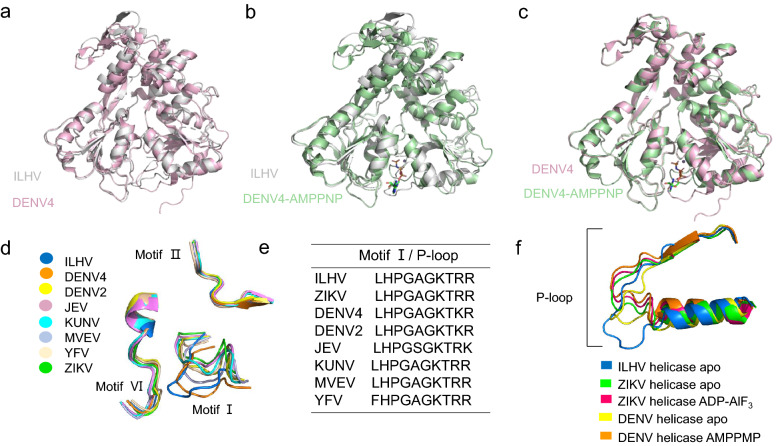
**a** Superimposition of the structures of ILHV helicase and the DENV helicase (PDB: 2JLQ). **b** Superimposition of the structures of ILHV helicase and DENV helicase in complex with AMPPNP (PDB:2JLR). **c** The structural comparison of the DENV helicase (PDB: 2JLQ) and DENV-helicase-AMPPNP complex (PDB:2JLR). **d** Comparison of motif I, II, and VI in the helicases of 8 flavivirus which are colored in indicated color codes. **e** Sequence alignment of ILHV helicase P-loop with other flavivirus helicases. **f** Structural superimposition of the P-loop of ILHV helicase with other flavivirus helicases

We superimposed the structure of ILHV helicase upon the structures of DENV4 helicase and ZIKV helicase in apo and AMPPNP/ATP bound forms and compared these structures. The overall structure of apo ILHV-helicase was highly similar to that of DENV4 apo and DENV4-helicase-AMPPNP complex with RMSD of 0.931 and 1.007 for Cα atoms, respectively (Fig. 2a, b). We also superposed the crystal structure of the apo form of DENV4 helicase with DENV4-helicase-AMPPNP-complex (Fig. 2c). Figure 2c showed the conformational change of DENV4 helicase upon ATP binding. ILHV helicase was more similar to DENV4 apo form than DENV4-helicase-AMPPNP-complex. These results demonstrated that ILHV helicase also need conformational change after binding ATP. Meanwhile, ILHV-helicase structure was found to be relatively conserved compared to the apo form of ZIKV and ZIKV-helicase-ATP complex with RMSD of 1.047 and 1.253, respectively (Additional file 1: Fig. S2a, b). ILHV helicase is evolutionarily closer to DENV4 than to ZIKV. The high conservation among the helicases of ILHV, DENV and ZIKV suggests the possibility for designing wide-spectrum inhibitors against all the flavivirus members.

Of note, motifs I, II, and VI were located at the borders of ILHV helicase domains I and II. These motifs might play an important role in combining the helicase with NTP and divalent metal ions. Superposition of the ILHV motifs I, II, and VI structures upon the other seven flaviviruses (DENV-2, DENV-4, JEV, KUNV, MVEV, YFV, and ZIKV) revealed that the structural conformation changes of motifs II and VI were relatively small (Fig. 2d). However, the P-loop (motif I) evinced a variety of flavivirus helicase structural conformations (Fig. 2d). We then compared the sequences of the P-loop of these viruses and the results showed that the sequences are highly conserved (Fig. 2e). In fact, the P-loop sequences of ILHV, KUNV, MVEV, and ZIKV were found to be identical. The P-loop sequences of DENV2, DENV4, JEV, and YFV were found to differ by one or two amino acid residues with those of ILHV, KUNV, MVEV, and ZIKV. The P-loop is essential for NTP binding and catalysis. Upon binding with RNA, helicases undergo major conformational changes, making the P-loop shifting toward the protein core and switching to a catalytically competent state [24, 25]. We also compared the P-loop of ILHV helicase in the apo form with the structures of ZIKV helicase and DENV4 helicase in apo and ADP/AMPPNP-bound forms (Fig. 2f). Relatively few differences were observed between the ILHV P-loop conformations, and the ZIKV and DENV4 apo forms, particularly compared to those observed with the ZIKV and DENV4 complexes. The P-loop of ILHV helicase was positioned closer to motif VI than other flavivirus helicases when not bound to NTP. It exhibited a “closed” conformation, which implies that the NTP binding pocket of ILHV helicase might need to undergo significant local rearrangements in transit to an active state.

### RNA binding of ILHV helicase

Replication is critical for the survival of flaviviruses. During flavivirus replication, the helicase protein is responsible for the ATP-dependent unwinding of the dsRNA’s intermediate structure. The ILHV helicase was well conserved across all flaviviruses (ZIKV, YFV, DENV, JEV, WNV, etc.) with the sequence identity of more than 60% (Additional file 1: Fig. S3). We speculated that the ILHV helicase domain also possessed three enzymatic activities. In the process of ILHV genome replication, two of the three enzymatic activities, nucleotide triphosphatase (NTPase) activity and RNA unwinding activity, are responsible for the translocation and unwinding of the replicative form. The third enzymatic activity, RTPase activity, functions to prime the genomic RNA strand which is newly synthesized for the capping of RNA by NS5, as speculated in the present study and in a report by Du Pont et al. [25].

**Fig. 3:**
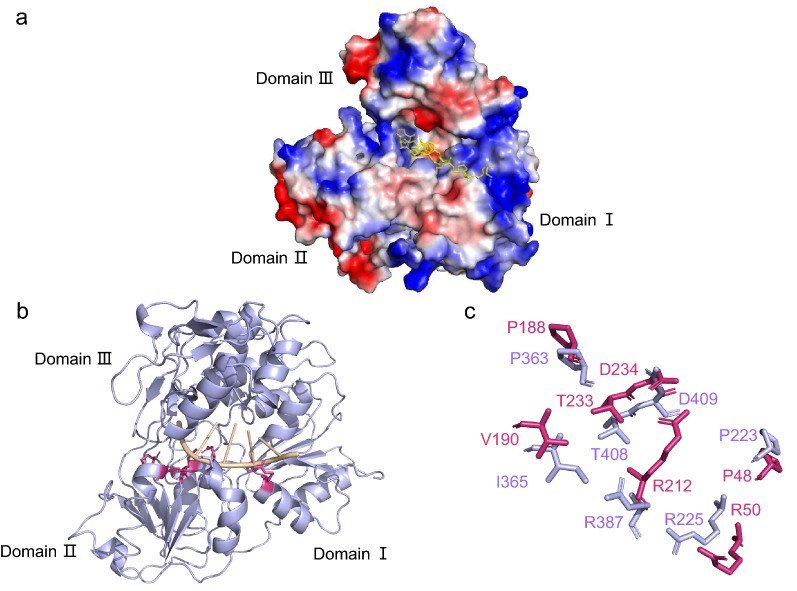
**a** The surface charge distribution and the RNA-binding site on the helicase of ILHV. The RNA is shown in yellow sticks. **b** A model of an ILHV-helicase-RNA complex. The RNA was modeled into the ILHV helicase structure by superimposition with the DENV4 helicase-RNA complex (PDB: 2jlv). Residues that were predicted to contact RNA are colored warm pink. The RNA is colored in wheat. **c** The comparison of residues which were predicted to contact RNA from ILHV helicase and DENV4 helicase

According to studies on other flaviviruses, the concave surface between ILHV helicase domains I and II can be deduced to bind with the ssRNA substrate. The 3′ end of ssRNA mainly connects with domain I, whereas the 5′ end binds to domain II. ILHV helicase domain III may be involved in binding the RNA-dependent RNA polymerase (RdRp) NS5 [24, 26]. In the ILHV helicase electrostatic surface potential model developed in the present study, a RNA binding favorable, positively charged cleft was found on the boundary of domains III and II to the boundary of domains III and I (Fig. 3a). In addition, ILHV helicase motifs Ia, IV, IVa, and V were likely responsible for RNA binding, translocation, and interdomain communication (Fig. 3b). Upon aligning the sequences of ILHV and DENV-4 (Additional file 1: Table S2), we observed that residue P48 of Motif Ia, P188 of Motif IV, and D234 of motif V interacted with the ssRNA ribose 2′-hydroxyl group. Meanwhile, residue R50 of Motif Ia, V190 of Motif IV, R212 of Motif IVa, and T233 of Motif V interacted with the ssRNA phosphate backbone. Almost all of these amino acids were highly conserved (Fig. 3c). These results reveal that a striking similarity exists between IHLV helicase and DENV, particularly in terms of the ssRNA recognition molecular details. They are also suggestive of an ancestral ssRNA recognition module that may have been incorporated by the flaviviridae during the course of evolution. Nevertheless, mutational studies are needed to further verify these putative interactions [24–26].

### NTPase activity sites of the ILHV helicase

Four conserved motifs, including motifs I, II, III, and VI, are responsible for ATP binding and hydrolysis. To gain further insight into the ILHV ATP hydrolysis process, we docked ATP and Mn^2+^ to the ILHV helicase and visualized the helicase-ATP-Mn^2+^ complexes using Pymol software (Additional file 1: Fig. S4a). On observing this ILHV helicase-ATP-Mn^2+^ complex, we noted that ATP-Mn^2+^ lied at the cleft between domains I and II and interacted with the P-loop. Based on the ATP and helicase interaction patterns, we clearly identified the amino acid residues which combined ATP in the active pocket. As shown in Additional file 1: Fig. S4a, the main residues in the active site were R26, R284, R287 and E110. The three arginine residues are critical for the stabilization of the ATP triphosphate. Residue E110, and the phosphate of ATP formed relatively strong electrostatic interactions with Mn^2+^. These were essential for stabilizing the Mn^2+^ in the active site. The apo form of ILHV-helicase and the ILHV-helicase-ATP-Mn^2+^ complex showed two states in the P-loop: a “closed” state and an “open” state. In the closed conformation, the P-loop was closer to motif VI and thus blocked the ATP-Mn^2+^-binding cleft; in the open conformation, the P-loop conformation was thus rearranged, resulting in its movement away from motif VI and the accommodation of ATP-Mn^2+^ (Additional file 1: Fig. S4b). We superimposed the ILHV-helicase-ATP-Mn^2+^ with the ZIKV- helicase-ATP-Mn^2+^ complex (Additional file 1: Fig. S4c). In the ZIKV helicase, K200, R459, and R462 interacted with the γ-phosphate of ATP, and the carbonyl oxygen of R462 also formed hydrogen bonds with the 3′-OH group of the ribose. The equivalents of these residues in ILHV helicase were K24, R284, and R287, respectively. It is worth mentioning that the R202 in ZIKV, which corresponds to R26 in ILHV helicase, formed a hydrogen bond with the N-7 atom of the adenosine to stabilize the adenosine. Mn^2+^ was accommodated by the side chains of E286 and T201, which corresponded to residues E110 and T25, respectively, in ILHV helicase. Q455 of ZIKV helicase accommodated a catalytic water and the nearby γ-phosphate, matching with the residue Q280 in ILHV helicase (Additional file 1: Fig. S4c). These residues were critical for ATP hydrolysis; a finding which aligns well with the molecular docking results (Additional file 1: Fig. S4c).

The most important residues within the ILHV helicase, R26, E110, and Q280, were mutated in molecular docking so as to determine their contribution in ATP hydrolysis (Additional file 1: Figs. S5a–d, S6). The molecular docking results showed that, after mutation, the hydrophobicity of ATP active site pocket of the ILHV helicase was increased, making the weaken interaction of ATP with this site (Additional file 1: Table S3). Comparing the binding energy of the three mutants and the wild-type ILHV helicase with ATP, we found that the binding energy of the mutated helicase was significantly reduced, indicating a poor combination ability of the mutated helicase with ATP (Additional file 1: Table S4). Notably, the binding energy of the mutant R26A with ATP was − 7.13 kcal/mol, a value significantly lower than that of the other mutants. This was mainly because the guanidine of the R26 could form strong hydrogen bond interaction with the phosphate of ATP. When arginine was mutated to alanine, the hydrogen bond interaction was unable to form (Additional file 1: Fig. S5b). After the mutation of residue E110, Mn^2+^ changed its initial position (Additional file 1: Fig. S5c). Because E110 could stabilize the metal ion Mn^2+^, after E110A mutation, Mn^2+^ could not stabilize at the original position and moved to the position close to the residue Q280, leading to electrostatic interaction with the residue Q280.

To further investigate the interactions between ATP, Mn^2+^, and the wild type or mutant helicases, we applied the 100-ns molecular dynamics simulations for the wild type/mutant ILHV helicase-ATP-Mn^2+^ complexes. The RMSD reflects complexes stability. A larger RMSD indicates higher stability. Additional file 1: Fig. S7a shows that the RMSD of ILHV helicase-ATP-Mn^2+^ complexes had slight fluctuations before 50 ns and remained stable in 50–70 ns, reflecting the consistent collision of the ligands with the active site in the protein pocket. The average RMSD of the complexes was 2.4 Å, indicating complexes fine stability and also demonstrating that the stable complexes did not elicit off-target effects in the molecular dynamics simulations analysis. The RMSD of the complexes exhibited obvious fluctuations between 70 and 100 ns. This was likely caused by the adaptive adjustments of ligands in the protein pocket through consistent dynamic balance. After the ligands being adjusted to a reasonable conformation, the complexes remained stable. RMSF reflects the conformational changes of each amino acid of the protein. We found that the conformational changes of amino acids around 25th, 70th, 145th, and 380th sites were relatively large (Additional file 1: Fig. S7b). This was mainly because these sites were located in the loop regions, which led to significant changes in the RMSF. On combining the results of the analyses of RMSD and RMSF of the complexes before and after mutation, it was found that mutant helicases stability exhibited little change. This suggested that these amino acid mutations would not have a significant effect on the protein conformation, but might change the ATPase activity of the ILHV helicase.

### ATPase activities of the wild-type ILHV helicase and the mutants

**Fig. 4:**
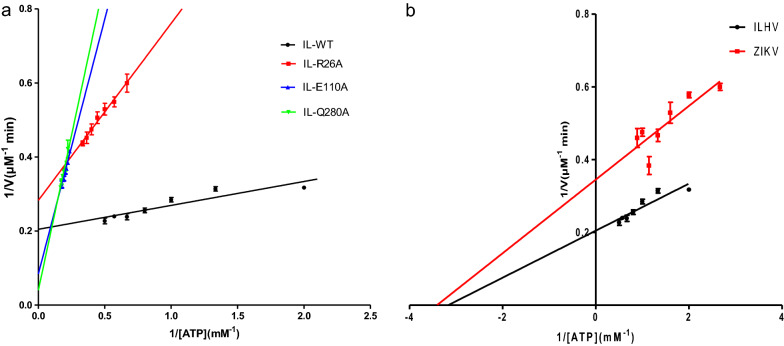
**a** Comparison of ATPase activity of the wild-type ILHV helicase, and three mutants (R26A, E110A, and Q280A). **b** Comparison of ATPase activity of the wild-type ILHV helicase and ZIKV helicase. The double-reciprocal plot was fitted according to the Michaelis–Menten equation

According to our molecular docking analyses, ILHV helicase residues R26A, E110A, and Q280A were found to be critical for ATP hydrolysis. Based on the sequence and structural alignments, these residues were also conserved between ILHV helicase and ZIKV helicase. They also played important roles in the binding of ATP, Mn^2+^ coordination, and nucleophilic attack [27, 28]. To confirm the contribution of the ATP hydrolysis associated residues, we constructed the R26A, E110A, and Q280A helicase protein mutants. The ATPase activities of these mutants were tested. Based on the Michaelis–Menten parameters, we fitted the data to present the double-reciprocal plot. Figure 4a shows the ATPase activity of wild-type and mutant ILHV helicases. The ATP hydrolysis kinetic parameters of both the wild-type and mutant ILHV helicases are presented in Additional file 1: Table S5. ILHV helicase, R26A, E110A and Q280A display the ATPase activity with K_cat_/K_m_ values of 3432.18, 446.71, 139.95, 110.27 M^−1^·S^−1^, respectively. The results indicated that R26A, E110A, and Q280A mutants greatly reduced the ATPase activity. Compared with the wild-type ILHV, the K_cat_/K_m_ values of E110A and Q280A reduced to less than 5% of that of wild-type ILHV. In addition, the K_cat_/K_m_ value of R26A was approximately 13% that of wild-type ILHV, this finding was consistent with the results of the molecular docking analysis (shown in Additional file 1: Table S4). Based on the ATPase activity assay, we concluded that R26, E110, and Q280 play critical roles in ILHV helicase catalytic activity. In addition, we measured the ATPase activity of ZIKV helicase with the same condition (Fig. 4b). Results indicated that the ZIKV helicase could hydrolyze ATP with a K_m_ value of 0.285 ± 0.069 mM and K_cat_ = 2.432 ± 0.250 S^−1^. This value was comparable to those of previous reports [27, 28]. Further, the K_m_/K_cat_ value of ZIKV was 8533.33 M^−1^·S^−1^. These results indicated that the ATPase activity of ILHV helicase was much lower than that of ZIKV helicase.

In addition, we compared the ATPase activity of ILHV helicase measured here with the ATPase activities of other flavivirus helicases previously reported (Additional file 1: Table S6). Results showed that the ILHV helicase hydrolyzed ATP with a K_cat_/K_m_ value of 3432.18 M^−1^·S^−1^ (Michaelis constant K_m_ = 0.317 ± 0.036 mM, turnover number K_cat_ = 1.088 ± 0.068 S^−1^S^−1^). This value was much lower than that of ZIKV, MVEV, DENV, and ALSV helicases [24, 27, 29, 30]. The difference of the helicase ATPase activity among different flaviviruses suggests that ILHV helicase has a diverse ATP binding ability, although it shows a conserved pattern in ATP hydrolysis compared to other flaviviruses.

### Inhibitors targeting the ILHV helicase

The NS3 helicase of flavivirus is a critical target for drug design. Currently, no specific drugs are available for flaviviruses. However, many studies regarding the inhibitors that target the helicase of flavivirus are underway. As for DENV, a novel small molecule inhibitor of DENV, ST-610 that targets the NS3 helicase, was found to inhibit the replication of DENV [31]. In addition, suramin could also inhibit the helicase activity of DENV [32]. For ZIKV, epigallocatechin-3-Gallate (EGCG), a green tea polyphenol, exerts a negative effect on ZIKV survival by inhibit the ZIKV NS3 helicase activity [33]. The compounds ST-610 and suramin were docked to ILHV helicase. Molecular docking results showed that the compounds have good binding effects with ILHV helicase with binding energies of − 7.58 kcal/mol and − 13.41 kcal/mol, respectively (Additional file 1: Table S7). The residues which connect with ST-610 include P188, R212, H309, V254, V190, S189, D427, and M267. Of note, the residues P188, R212, D427 formed strong hydrogen bonds with ILHV helicase, thus significantly contributing to the stabilization of small molecules in the active pocket (Additional file 1: Fig. S8a). Suramin formed strong interactions with residues N428, C426, R191, K213, T304, P335, A276, P144, P256, and V190. The two ends of the sulfonic acid groups of suramin formed strong hydrogen bond interactions with N428, C426, R191, K213, T304, and P335 (Additional file 1: Fig. S8b). In summary, ST-610 and suramin were observed to formed stable complexes with ILHV helicase. Thus they have the potential to function as the ILHV helicase inhibitors.

## Discussion

The crystal structure of ILHV helicase protein was solved in this study. Sequence alignment and structural comparison indicated that the overall ILHV helicase structure is conserved among flavivirus helicases. ILHV helicase exhibits well conserved residues for RNA binding in some flaviviruses like DENV-4 and ZIKV. Moreover, the mode of helicase binding of ATP and Mn^2+^ is shared between viruses belonging to the Flavivirus genus [22, 26–28, 34]. It is thus conceivable that the mechanism of ATP hydrolysis of that drives the unwinding of RNA is also shared. After consulting the literature, we found that some small molecule inhibitors of DENV, like ST-610 and suramin which target the NS3 helicase of DENV [32], could also inhibit the helicase activity of ILHV. We docked these two compounds to ILHV helicase, and found that both of the two compounds could form stable complexes with ILHV helicase (Additional file 1: Fig. S8). These results suggest the possibility of designing wide-spectrum inhibitors targeting all flaviviruses [35].

The P-loop, which is formed by the secondary structure of the Motif I, allows the residues within the motif to bind to NTP. Thus, the P-loop is a very important structure for ILHV and worth noting [26]. We compared the sequences of the P-loops of different flaviviruses and found that the sequences were highly conserved. The P-loop sequences of ILHV, KUNV, MVEV, and ZIKV were identical, while the P-loop sequences of DENV2, DENV4, JEV, and YFV differed from those of ILHV, KUNV, MVEV, and ZIKV by one or two amino acid residues. Although the residues in the P-loops are highly conserved among flaviviruses, the P-loop conformation is variable [36]. In fact, a single-point mutation of a P-loop residue and the changed conformation of P-loop can result in altered nucleotide binding, specificity, and accessibility, as well as in catalytic variation in the energy producers [37]. In addition, the structural transition of ILHV helicase from the apo form to the ATP binding form can induce significant conformational changes of the P-loop. When the P-loop is closer to the ATP binding cleft, the ATP-binding pocket exhibits a closed state. In turn, when ATP and Mn^2+^ bind to the ATP binding cleft, the structure of helicase undergoes a conformational rearrangement. The ATP-binding pocket would change to an open state, rendering it more catalytically active. To achieve optimal ATPase activity, the P-loop shifts toward the protein core [24, 25]. In the present study, the P-loop of apo ILHV helicase was located closer to the motif VI than other flaviviruses, exhibiting a more closed state (shown in Fig. 2d).

Our molecular docking results also support the viewpoint that the ATP hydrolysis function of ILHV helicase requires a highly dynamic interplay between alternative conformations; with P-loop structural transitions being essential for function. Therefore, this flexible P-loop may constitute a valuable target for blocking the replication cycle of flaviviruses using antiviral compounds that stabilize the helicase molecule in a single conformation. This thereby interferes with its ATPase activity. To gain functional insights into the ILHV helicase, we performed the ATPase assay and compared the ATPase activity of ILHV helicase with those of DENV, ZIKV, and MVEV. Results indicated that the ATPase activity of ILHV was lower than that of other flaviviruses. These findings suggest that flaviviruses could have a conserved pattern of ATP hydrolysis in tandem with variable ATP binding abilities to adapt to their individual replication.

Prior reports have shown that the ATPase activity of helicase is related to the infectivity and pathogenesis of flaviviruses. One study constructed the mutants of DENV2 helicase and the results indicated that the mutagenesis exerted different effects on enzyme activity and replication of the virus. Some of the mutants showed increased ATPase activity and helicase activity, and also significantly increased virus titer [38]. Another study constructed three mutants of hepatitis E virus helicase proteins. Compared with the wild-type (WT) protein, the ATPase activities of the mutants L1110F and V1120I decreased significantly and had a negative effect on virus replication [39]. Langevin et al. [40] found in the American crow (AMCR) model that the effect of WNV NS3-249 amino acid mutation on the ATPase activity appeared to be functionally corresponding to the virulence. Swarbrick et al. [41] reported that three mutants (D290A, R538A, and G540P) of DENV helicase decreased the RNA replication rates, consistent with decreased extracellular viral RNA levels. At the same time, the BHK-21 plaque assay showed smaller plaques formed by the mutants than the WT. In addition, immunofluorescent staining of the transfected cells showed a much lower NS3 mutant infection percentage compared to WT. These studies may give a reason for the lower ATPase activity of ILHV helicase.

## Conclusion

We determined a high-resolution structure of ILHV helicase. Our molecular docking results and biochemical characterizations of ILHV helicase revealed critical candidate targets for designing inhibitors targeting the ATP binding tunnel. The study may provide a support for human battles against the present and possible future outbreaks of flavivirus infections across the world.

## Methods

### Cloning, protein expression and purification

The full-length NS3 helicase gene (1338 bp) of Ilheus virus was cloned in the vector pET.32M.3C and expressed in the *E. coli BL21 (DE3)* cells (Solarbio, China). The cells were cultured in the LB medium containing 100 μg/ml ampicillin at 37 ℃ in a shaker incubator. Then, the expression of helicase protein was induced by 1 mM isopropyl-β-d-thiogalactopyranoside (IPTG) at 16 ℃ for 16 h. Finally, the cells were centrifuged at 4000 rpm for 20 min and resuspended in buffer A (20 mmol/L Na_2_HPO_4_, 0.5 mol/L NaCl, 20 mmol/L imidazole, pH 8.0). High pressure homogenization was used to lyse the cells and the lysate was centrifuged at 15,000 rpm for 1 h at 4 ℃ to harvest the supernatant. In brief, ILHV helicase protein was purified by Ni Sepharose (GE) affinity chromatography, anion-exchange chromatography, and size exclusion chromatography. The purification of ILHV helicase protein was as followed. Before the purification of the supernatant, Ni Sepharose (GE) affinity chromatography mediums were equilibrated with buffer A. The proteins without His-tag were eluted with buffer A and the target proteins were eluted with buffer B (20 mmol/L Na_2_HPO_4_, 0.5 mol/L NaCl, 250 mmol/L imidazole, pH 8.0). The collection was incubated with PreScission Protease overnight at 4 ℃ for about 12 h. After ultrafiltration and dilution in buffer C (50 mmol/L Hepes, 50 mmol/L NaCl, 5% glycerol), the collection was injected into a HiTrap™ SP HP 5 ml column and eluted with buffer C and buffer D (50 mmol/L Hepes, 1 mol/L NaCl, 5% glycerol, pH 6.8), using a liner NaCl concentration gradient. The concentrated target proteins were loaded onto the Superdex™ 200 Increase 10/300 GL column equilibrated with buffer E (10 mmol/L Tris–HCl, 150 mmol NaCl, 5 mmol DTT, 5% glycerol). Finally, the ILHV helicase proteins were collected and adjusted the concentration to 5 mg/ml and 10 mg/ml. The cloning, expression, and purification of ZIKV helicase_180−617_ were performed according to the previous study [27].

### Site-directed mutagenesis of ILHV helicase

Three mutants of ILHV helicase, including R26A, E110A, and Q280A, were constructed by the Mut Express MultiS Fast Mutagenesis Kit V2 (Vazyme, Nanjing, China). Briefly, the wild-type plasmid was amplified by PCR with different mutagenic primers for the mutants R26A, E110A, and Q280A. All desired mutants were confirmed by sequencing. The expression and purification of the three mutants were the same with the wild-type.

### ATPase activity assay

The ATP activity assay was conducted using the ATPase/GTPase Activity Assay Kit (MAK113; Sigma‑Aldrich; Merck KGaA, Darmstadt, Germany). In the 96-well plate, 10 μl ILHV helicase protein solution at a concentration of 300 nM or 10 μl ILHV helicase protein solution at a concentration of 80 nM were preincubated in the 20 μl assay buffer (40 mM Tris, 80 mM NaCl, 8 mM MgAc_2_, 1 mM EDTA, pH 7.5). The reaction was carried out by adding 10 μl ATP at different concentrations for 25 min at 25 ℃. By adding 200 μl reagent buffer, the reaction was terminated. After incubating with reagent buffer for 30 min at room temperature, the absorbance was measured by SpectraMax iD3 multi-function reader (Molecular Devices, USA) at 620 nm. The K_m_ and K_cat_ of the enzyme were obtained from a double-reciprocal plot which was fitted according to the Michaelis–Menten equation with the GraphPad Prism Software (GraphPad, La Jolla, CA, USA). The ATPase activity of mutants R26A, E110A, and Q280A were also measured with the same condition.

### Crystallization

Crystals of Ilheus virus helicase were obtained at concentration of both 5 mg/ml and 10 mg/ml at 16 ℃ by the sitting drop. The precipitant condition is 0.1 M tris hydrochloride, pH 8.5, 0.2 M ammonium sulfate. Crystals were cryoprotected using the mixture of crystallization buffer and 25% glycerol. Then, the crystals suitable for X-ray experiments were flash-frozen by immersing in liquid nitrogen.

### Data collection and processing

The X-ray diffraction data were collected at Shanghai Synchrotron Radiation Facility (SSRF) beamline BL19U1 [27]. All the diffraction data were indexed, integrated and processed by HKL3000 software [42]. Additional file 1: Table S1 shows the data collection and processing details.

### Structure solution, refinement and analysis

The structure of ILHV helicase was solved by molecular replacement using PDB 2WV9 as a research model. PHENIX [43, 44] was used to refine the model and the model was manually constructed by Coot [45]. Finally, the positional and isotropic atomic displacement parameters were refined. PyMOL (http://www.pymol.org/) [24] was used to perform the figures. CCP4 was used to compare the structures and calculate RMSD [46].

### Molecular docking

The structures of ATP, Mn^2+^, ST-610, and Suramin for molecular docking are available from PubChem Database (https://pubchem.ncbi.nlm.nih.gov/) and are imported in the Schrödinger Maestro software to perform hydrogenation, structure refinement, and energy minimization. The structure of ILHV helicase was also processed in the Schrödinger Maestro software to remove crystal water, add the missing hydrogen atoms, restore the missing information of bonds, repair the lacking peptides, minimize the energy and optimize the structure [47, 48]. The process and optimization of virtual screening were completed by the Glide module in Schrödinger Maestro software. The Protein Preparation Wizard module was used to process the protein. We preprocessed, refined, and minimized the helicase protein and prepared ATP/Mn^2+^, ST-610, and Surmain according to the default setting in LigPrep module. When screening in the Glide module, we imported the prepared ILHV helicase and predicted its active sites and used SP for molecular docking.

### Molecular dynamics (MD) simulation

Desmond version 2020 was used to conduct MD stimulations of the wild type and mutant ILHV helicase- ATP/Mn^2+^ complexes. We used the force field OPLS3e to carry out the MD stimulation and the TIP3 water model for the solvation of the system. We used the SHAKE algorithm to restrain the geometric of water molecules, bond lengths, and angles of heavy atoms. Periodic boundary conditions and particle grid Ewald were used to simulate the continuous system and maintain the long-range electrostatic interactions, respectively. In the later stage, the time range was 100 ns with a time step of 1.2 fs and the track record was performed every 10 ps. A total of 1000 frames were recorded. We calculated the root mean squared deviation (RMSD) of the main chain atoms and conducted graphical analysis to obtain the characteristics of the wild type/mutant ILHV helicase-ATP/Mn^2+^ interactions. By calculating the root mean squared fluctuations (RMSF) of individual residues, we could analyze the significant conformational transformations between the initial and dynamic states of the residues.

## Supplementary Information

**Additional file 1: Table S1.** Data collection and refinement statistics. **Table S2.** Structure-based sequence alignment of ILHV and DENV-4. **Table S3.** The binding site for ILHV helicase before and after mutation. **Table S4.** The target protein docking results for ATP and Mn2+. **Table S5.** Summary of the ATPase activities of the ILHV helicase mutants. **Table S6.** The ATPase activity comparison of different flaviviruses. **Table S7.** The docking results for ST-610 and Suramin with ILHV helicase. **Figure S1.** Purification of ILHV helicase using gel filtration chromatography. **Figure S2.** Superimpositions of the crystal structure of ILHV helicase with different forms of ZIKV helicase. **Figure S3.** Structural alignment of the helicase domains of different flaviviruses. **Figure S4.** The NTP hydrolysis site and comparison with ZIKV of the ILHV helicase. **Figure S5.** The binding mode of ATP, Mn2+ with the wild-type ILHV helicase and three mutants. **Figure S6.** The overlap structures of three mutants before and after mutation. **Figure S7.** The RMSD plot and RMSF plot during molecular dynamics simulations of protein with ATP. **Figure S8.** The binding modes of ILHV helicase with ST-610 and Suramin.

## Data Availability

All data generated or analyzed during this study are included in this published article and its supplementary information files.

## Abbreviations

ILHV: Ilheus virus
DENV: Dengue virus
YFV: Yellow fever virus
ZIKV: Zika virus
CPCV: Cacicapore virus
BUSV: Bussuquara virus
ROCV: Rocio virus
SLEV: Saint Louis encephalitis virus
SF2: Superfamily 2
KUNV: Kunjin virus
MVEV: Murray Valley encephalitis virus
JEV: Japanese encephalitis virus
WNV: West Nile virus
NS3: Nonstructural protein 3
NS5: Nonstructural protein 5
NTPase: Nucleoside triphosphate hydrolase
RTPase: RNA triphosphatase
RdRp: RNA-dependent RNA polymerase
RMSD: Root mean squared deviation
RMSF: Root mean squared fluctuation
MD: Molecular dynamics
