# Supplementary material for: Crystal structure of the Ilheus virus helicase: implications for enzyme function and drug design
Source: Cell Biosci. 2022 Apr 15;12:44. doi: 10.1186/s13578-022-00777-8 (PMC9012436; doi:10.1186/s13578-022-00777-8)
Supplement: Supplementary file 1 — Additional file 1: Table S1. Data collection and refinement statistics. Table S2. Structure-based sequence alignment of ILHV and DENV-4. Table S3. The binding site for ILHV helicase before and after mutation. Table S4. The target protein docking results for ATP and Mn2+. Table S5. Summary of the ATPase activities of the ILHV helicase mutants. Table S6. The ATPase activity comparison of different flaviviruses. Table S7. The docking results for ST-610 and Suramin with ILHV helicase. Figure S1. Purification of ILHV helicase using gel filtration chromatography. Figure S2. Superimpositions of the crystal structure of ILHV helicase with different forms of ZIKV helicase. Figure S3. Structural alignment of the helicase domains of different flaviviruses. Figure S4. The NTP hydrolysis site and comparison with ZIKV of the ILHV helicase. Figure S5. The binding mode of ATP, Mn2+ with the wild-type ILHV helicase and three mutants. Figure S6. The overlap structures of three mutants before and after mutation. Figure S7. The RMSD plot and RMSF plot during molecular dynamics simulations of protein with ATP. Figure S8. The binding modes of ILHV helicase with ST-610 and Suramin. [file 13578_2022_777_MOESM1_ESM.docx]

**Supplementary material**

**Crystal structure of the Ilheus virus helicase: implications for enzyme function and drug design**

De-Ping Wang^1, *^, Mei-Yue Wang^1, *^, Yong-Mei Li^1^, Wen Shu^1^, Wen Cui^2^, Fang-Ying Jiang^1^, Xin Zhou^1, #^, Wen-Ming Wang^3, #^, Ji-Min Cao^1, #^

1. Key Laboratory of Cellular Physiology at Shanxi Medical University, Ministry of Education, and the Department of Physiology, Shanxi Medical University, Taiyuan, China

2. Institute of Life Sciences, Chongqing Medical University, Chongqing, China

3. Institute of Molecular Science, Shanxi University, Taiyuan, China

^*^ These authors contributed equally to this work.

^#^Corresponding Authors. Leading contact: Ji-Min Cao (email: caojimin@126.com)

**Supplementary Tables**

**Table S1.** Data collection and refinement statistics

| Data | ILHV helicase |
| --- | --- |
| **Data Collection** |  |
| X-ray Source | SSRF beamline BL18U1 |
| Wavelength (Å) | 0.97915 Å |
| Space group | *P4_1_2_1_2* |
| Unit cell a,b,c,α,β,γ (Å) | 112.2, 112.2, 76.5, 90.0, 90.0, 90.0 |
| Resolution range (Å) | 50.0-1.75 (1.78-1.75) ^a^ |
| Unique reflections | 49792 (2397) ^a^ |
| Completeness (%) | 99.9 (97. 6) ^a^ |
| Redundancy | 11.3 (9.4) ^a^ |
| *I/σI* | 43.3 (6.3) ^a^ |
| *R*_merge_ ^b^ | 0.071 (0.293) ^a^ |
|  |  |
| **Refinement** |  |
| Resolution range (Å) | 31.59-1.75 (1.81-1.75) ^a^ |
| No. of reflections (working/test) | 49652/4891 |
| *R*_work_/*R*_free_ ^c^ | 0.168/0.206 (0.188/0.243) ^a^ |
| Number of atoms |  |
| Protein  Water  ligand | 3663  615  14 |
| B-factors |  |
| Protein | 20.16 |
| Water  ligands | 32.66  35.22 |
| r.m.s. deviations |  |
| Bond lengths (Å) | 0.015 |
| Bond angles (°)  Ramachandran Plot ^d^  (% favored/allowed/outliers) | 1.88  97.92/2.08/0.00 |

^a^ Values in parentheses are the statistics for the highest resolution shell.

^b^ R_merge_ =Σ_hkl_Σ_j_**|I**_hkl_ −**I**_hkl_(J)**|/**Σ_hkl_Σ_j_**|I**_hkl_(J)**|**. **I**_hkl_(J) and **I**_hkl_ represent the jth and mean intensity of reflection hkl.

^c^ R_work_ =Σ_hkl_**||F**_obs_**|**-**|F**_calc_**||/|F**_obs_**|.** **F**_obs_ and **F**_calc_ represent the observed and calculated structure factors, respectively. R_free_ is the R factor calculated with 10 % of unique reflections as the test set.

^d^ The values are reported by PHENIX.

**Table S2.** Structure-based sequence alignment of ILHV and DENV-4

|  | Ⅰ | Ⅰa | Ⅱ |
| --- | --- | --- | --- |
| **DENV4-NS3**  **ILHV-NS3** | LHPGAGKTKR  LHPGAGKTRR | LAPTRVVAAE  LAPTRVVAAE | VMDEAHFT  VMDEAHFT |
|  | Ⅲ | Ⅳ | Ⅳa |
| **DENV4-NS3**  **ILHV-NS3** | MTATP  MTATP | VMFVPSI  VWFVPSV | IQLSRKTF  IQLNRKSY |
|  | Ⅴ | Ⅵ | Function  ATP binding and hydrolysis  Ribose 2’-OH  Linker region  Phosphate backbone |
| **DENV4-NS3**  **ILHV-NS3** | FVVTTDISEMGAN  FVITTDISEMGAN | QRRGRIGRNPAQ  QRRGRIGRDPTQ |  |

The region residues related to ATP binding and hydrolysis, interdomain communication, binding of the ribose 2’-hydroxyl of RNA or backbone phosphate are colored in yellow, green, cyan, and magenta, respectively.

**Table S3.** The Binding site for ILHV helicase before and after mutation

| Target | Site | ATP binding | Mn^2+^ | Combination  Type |
| --- | --- | --- | --- | --- |
| ILHV helicase | R26 | + | - | Hydrogen bond |
| ILHV helicase | E110 | + | + | Electrostatic interaction |
| ILHV helicase | Q280 | - | - | Electrostatic interaction |
| ILHV helicase | R26A | - | - | Hydrophobic interaction |
| ILHV helicase | E110A | - | - | Hydrophobic interaction |
| ILHV helicase | Q280A | - | - | Hydrophobic interaction |

**Table S4.** The target protein docking results for ATP and Mn^2+^

| Target | Compound | Binding Energy (kcal/mol) | Combination Residues |
| --- | --- | --- | --- |
| ILHV helicase | ATP | -10.80 | R26, R287,  R284, E110 |
| ILHV helicase-R26A | ATP | -7.13 | K24, R287,  R284, E110 |
| ILHV helicase-E110A | ATP | -8.36 | R26, R287,  R284, Q280 |
| ILHV helicase -Q280A | ATP | -8.92 | R26, R287,  R284, G21 |

Note: Binding energy function


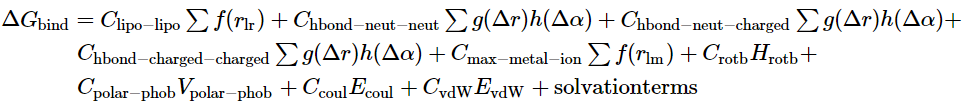


**Table S5.** Summary of the ATPase activities of the ILHV helicase mutants

| Mutation | K_m_ (mM) | k_cat_ (S^-1^) | k_cat_/k_m_ (M^-1^·S^-1^) |
| --- | --- | --- | --- |
| None* | 0.317±0.036 | 1.088±0.068 | 3432.18 |
| R26A | 1.764±0.179 | 0.788±0.049 | 446.71 |
| E110A | 22.666±15.340 | 3.172±1.531 | 139.95 |
| Q280A | 19.370±9.559 | 2.136±0.864 | 110.27 |

The ATPase assay was conducted by the ATPase/GTPase Activity Assay Kit (MAK113, Merck KGaA, Darmstadt, Germany)with 75nM ILHV helicase in the 40 μl reaction system. The Michaelis-Menten equation was used to fit the results. From the double-reciprocal plot processed by GraphPad Prism software, we obtained the k_m_ and k_cat_ values of the enzyme. *Wild type.

| Flavivirus | K_m_ (mM) | K_cat_ (S^−1^) | K_cat_/K_m_ (M^−1^·S^−1^) |
| --- | --- | --- | --- |
| ILHV | 0.317±0.036 | 1.088±0.068 | 3432.18 |
| ZIKV | 0.285±0.069 | 2.432±0.250 | 8533.33 |
| MVEV*^a^* | 0.38±0.03 | 5.3 | 13947.37 |
| DENV*^b^*  ALSV*^c^* | 0.30  0.055±0.008 | 5.8  0.61±0.04 | 19333.33  11090.91 |

**Table S6.** The ATPase activity comparison of different flaviviruses

^a^ Mancini EJ, Assenberg R, Verma A, Walter TS, Tuma R, Grimes JM, et al. Structure of the Murray Valley encephalitis virus RNA helicase at 1.9 Angstrom resolution. Protein Sci. 2007;16(10):2294-300.

^b^ Xu T, Sampath A, Chao A, Wen D, Nanao M, Chene P, et al. Structure of the Dengue virus helicase/nucleoside triphosphatase catalytic domain at a resolution of 2.4 A. J Virol. 2005;79(16):10278-88.

^c^ Gao X, Zhu K, Wojdyla JA, Chen P, Qin B, Li Z, et al. Crystal structure of the NS3-like helicase from Alongshan virus. IUCrJ. 2020;7(Pt 3):375-82.

| Target | Compounds | Structure | Docking Score (kcal/mol) | Combination Type |
| --- | --- | --- | --- | --- |
| ILHV helicase | ST-610 | 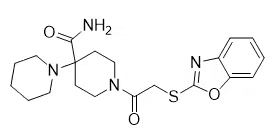 | -7.58 | Hydrogen bonds,  Hydrophobic interactive,  π-stacking |
| ILHV  helicase | Suramin | 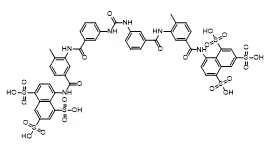 | -13.41 | Hydrogen bonds,  Hydrophobic interactive |

**Table S7.** The docking results for ST-610 and Suramin with ILHV helicase

**Supplementary Figures and Legends**


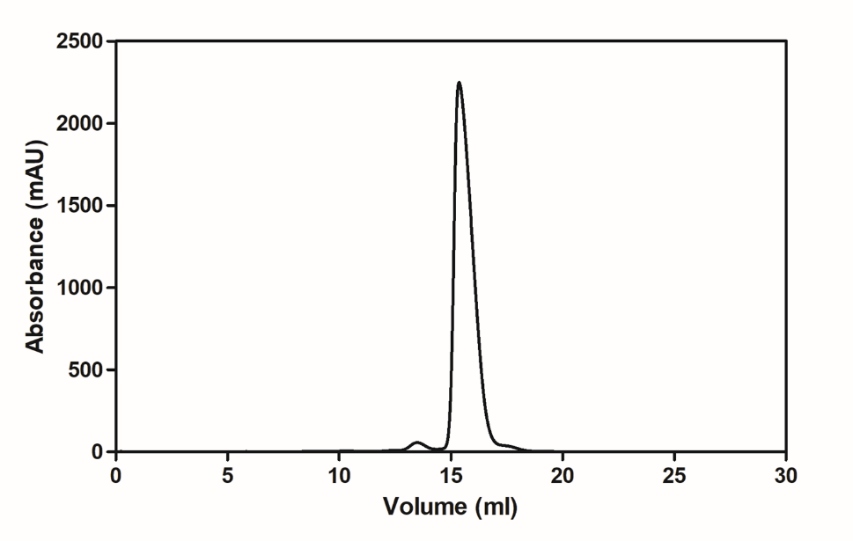


**Figure S1.** Purification of ILHV helicase using gel filtration chromatography (Superdex^TM^ 200 Increase 10/300 GL column).


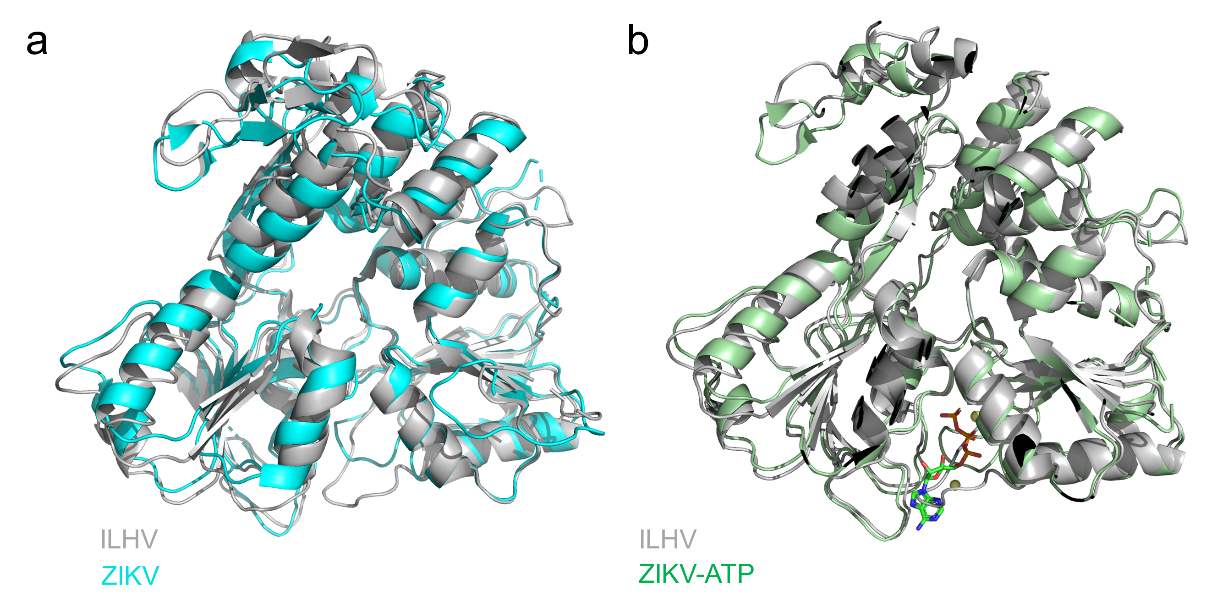


**Figure S2.** **a**, Superimposition of the crystal structures of ILHV helicase and the apo form of ZIKV helicase (PDB: 5jmt). ILHV helicase is colored in grey and ZIKV helicase is colored in light blue; **b**, The structural comparison of ILHV helicase and ZIKV helicase-ATP complex (PDB: 5K8I), which are colored in grey and green, respectively.


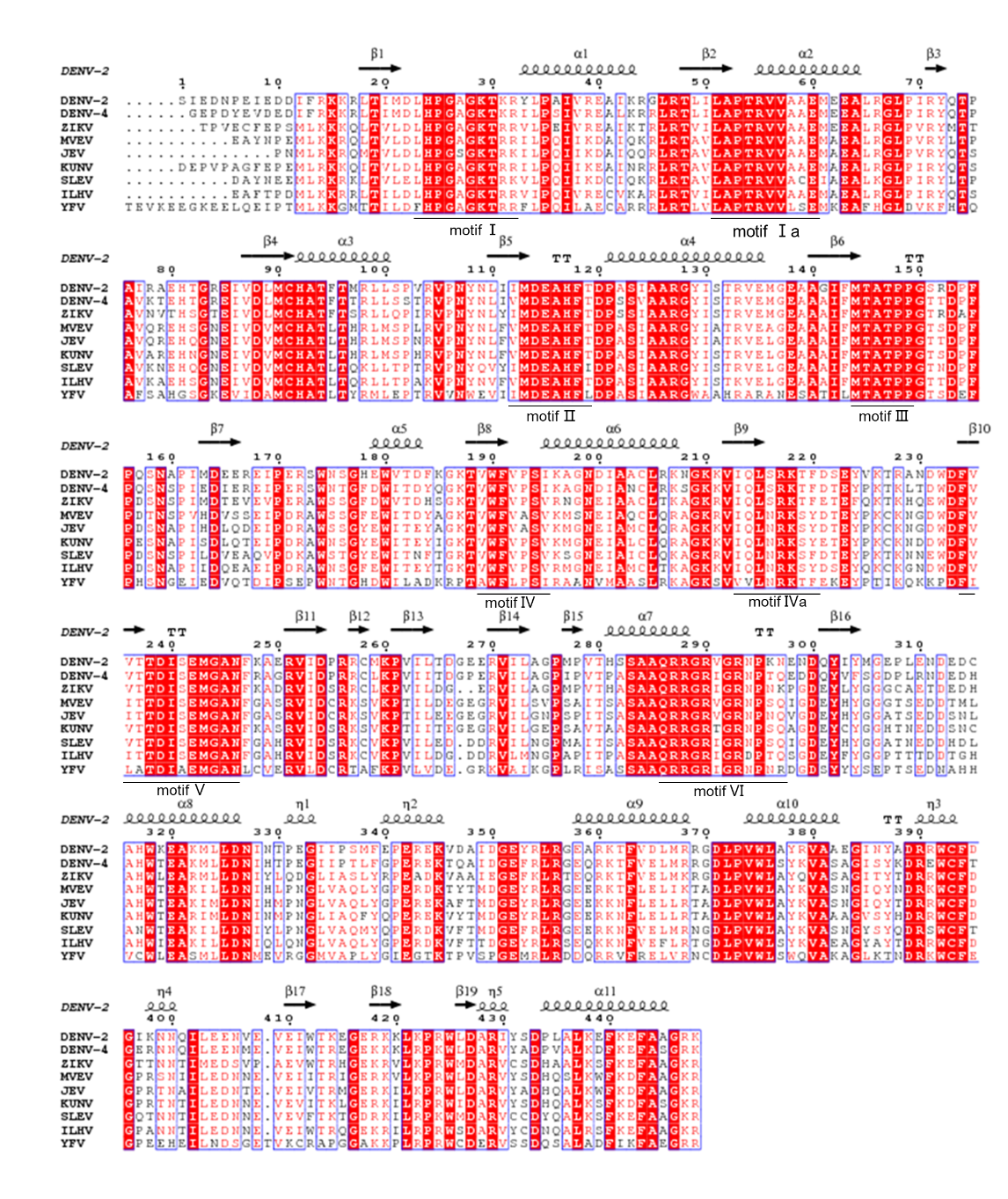


**Figure** **S3.** Structural alignment of Flavivirus helicase domain of DENV-2 (GenBank: AGX15365.1), DENV-4 (GenBank: AVW85696.1), ZIKV (GenBank: AQS26822.1), MVEV (GenBank: AHF27227.1), JEV (GenBank: AAQ73514.1), KUNV (GenBank: AOS89787.1), SLEV (GenBank: QJD26115.1), ILHV (GenBank: AGJ84083.1), and YFV (GenBank: AWB15005.1).


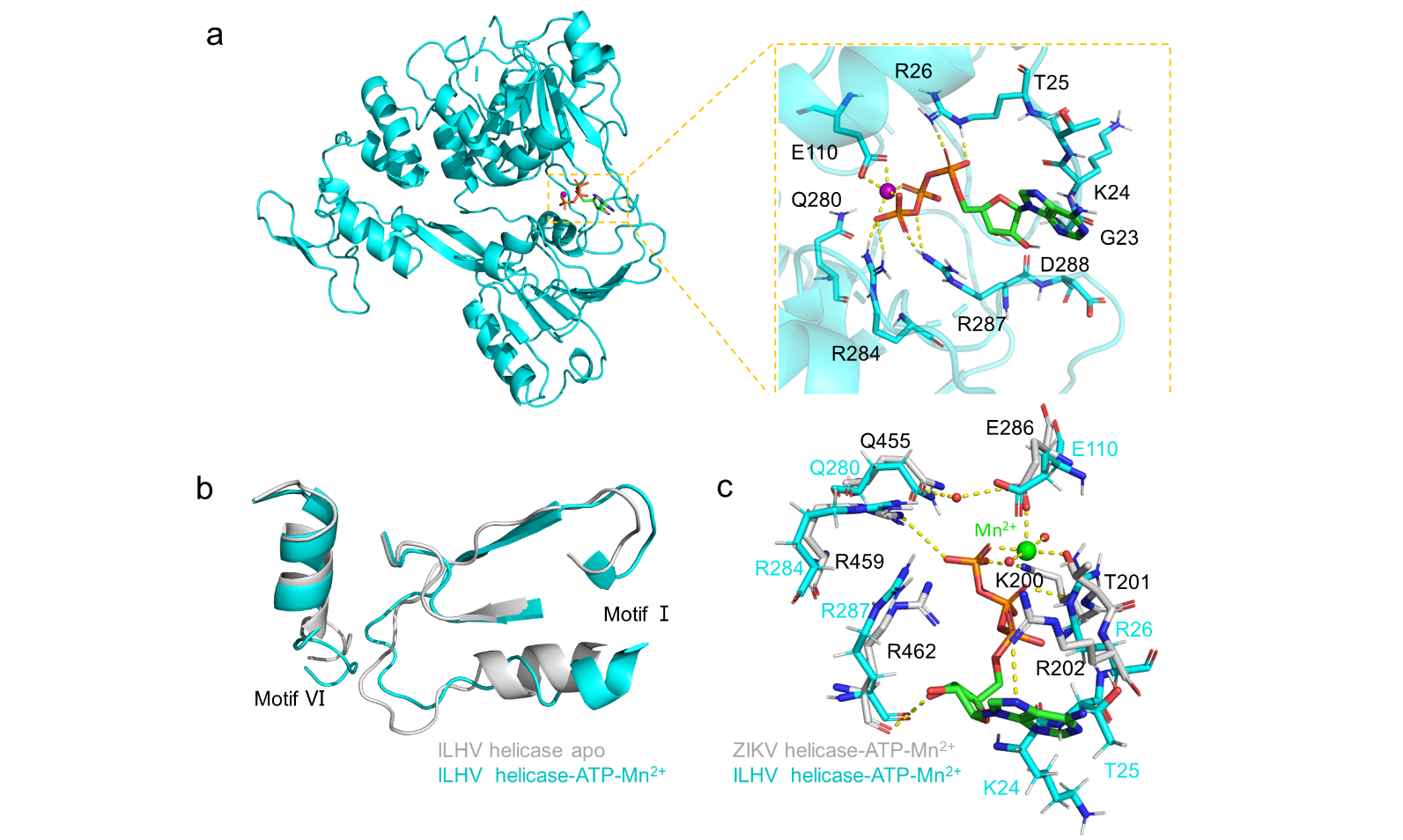


**Figure S4.** **a**, The NTP hydrolysis site of the ILHV helicase. **b**, The comparison of the motif Ⅰ, Ⅵ of ILHV helicase and ILHV helicase-ATP-Mn^2+^ complex. **c**, The structural comparison of the ILHV-helicase-ATP-Mn^2+^ with the ZIKV- helicase-ATP-Mn^2+^ complex.


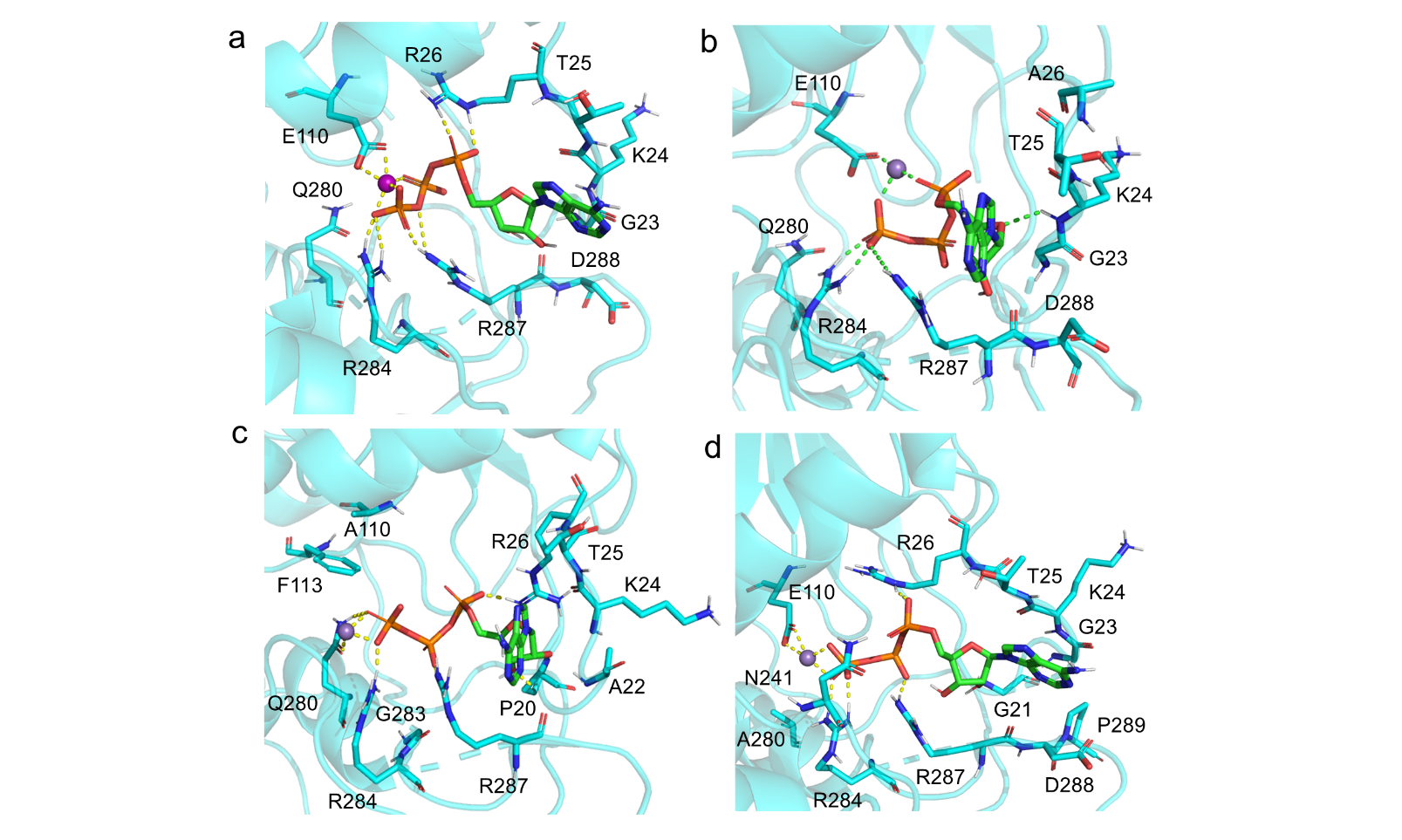


**Figure S5.** The binding mode of ATP, Mn^2+^ with the wild-type ILHV helicase (**a**) and the mutants R26A (**b**), E110A (**c**), and Q240A (**d**). The residues which combined with ATP and Mn^2+^ are annotated in the corresponding position.


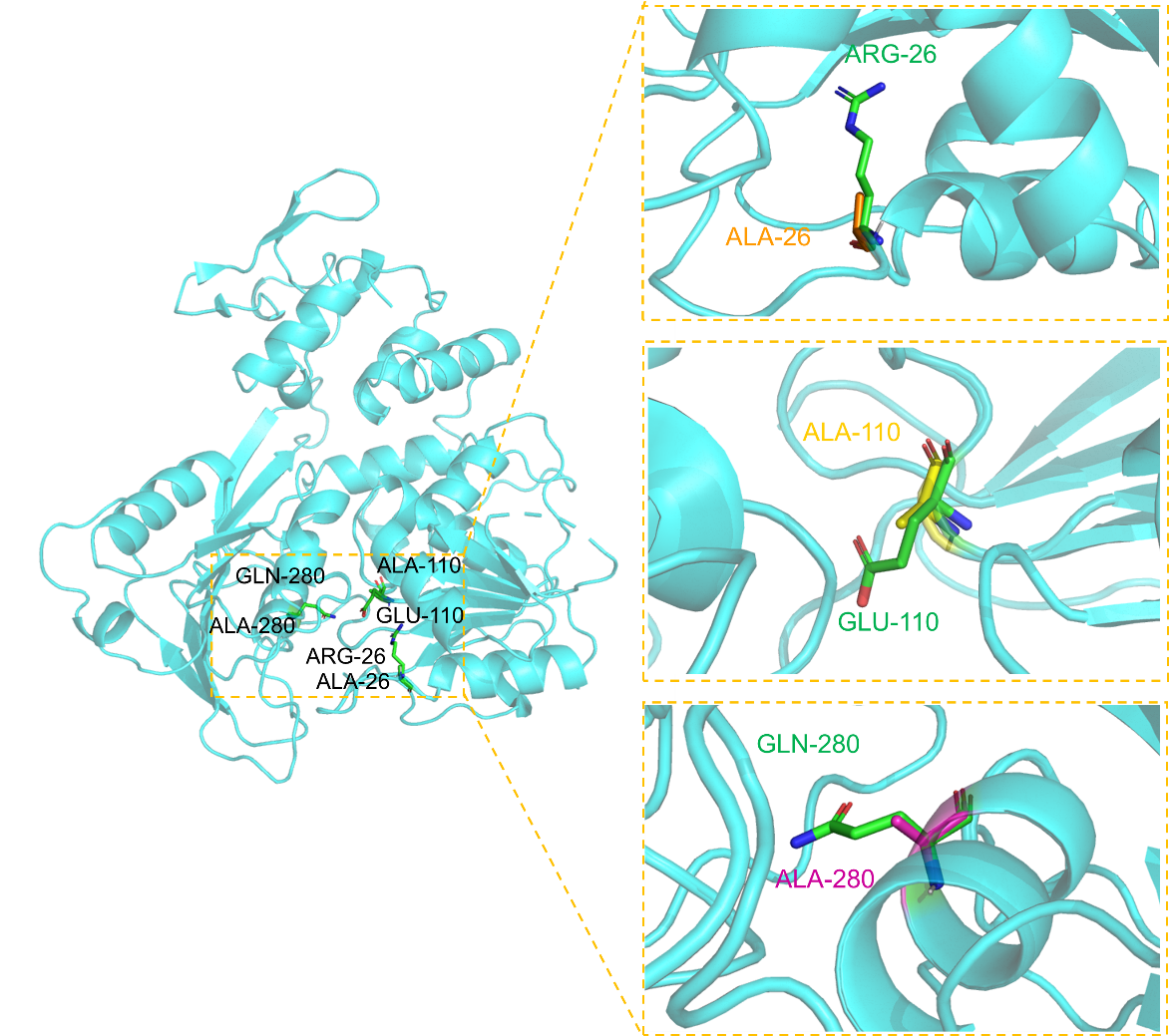


**Figure S6**. The overlap structures of three mutants R24A, E110A, and Q280A before and after mutation.


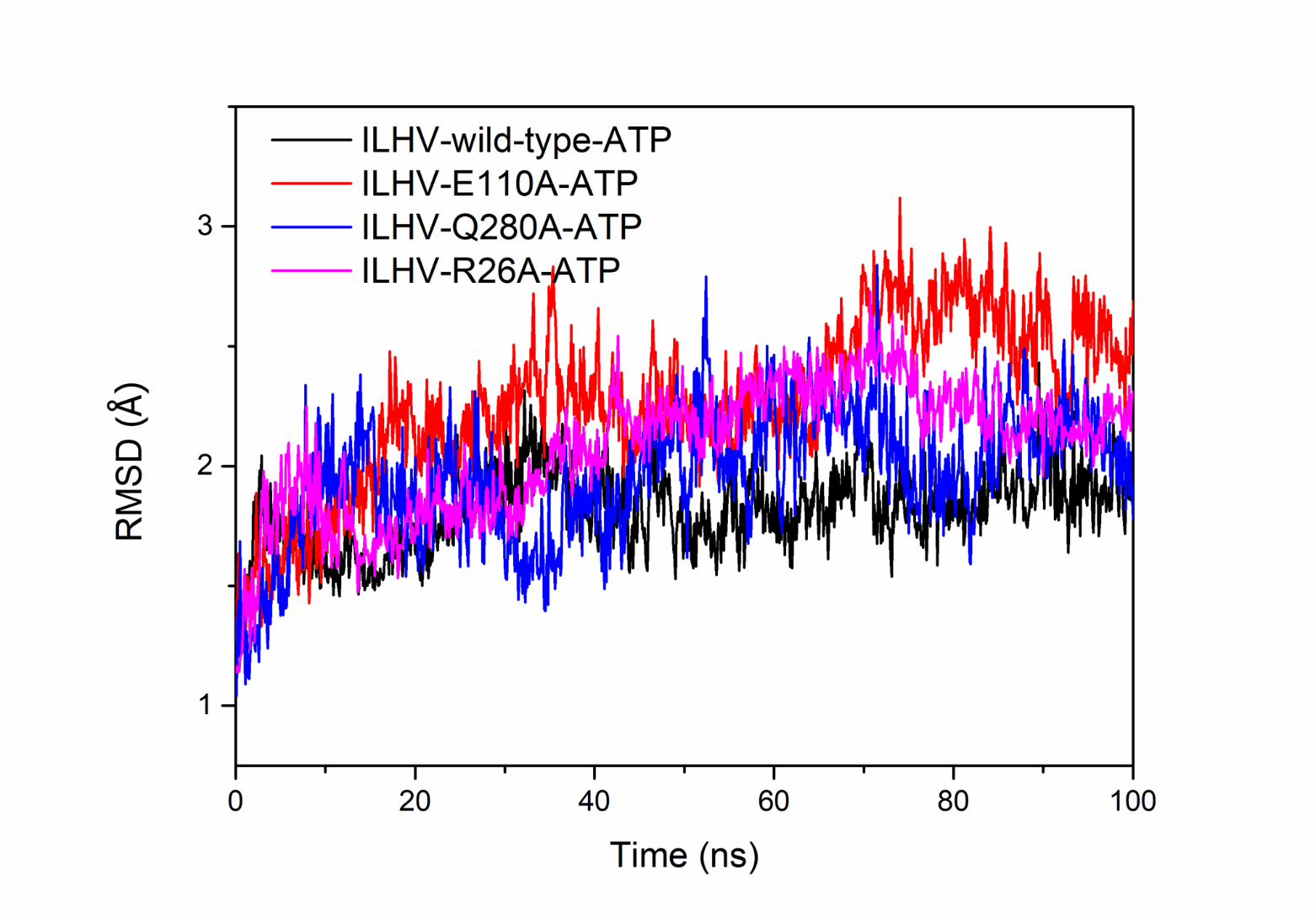


a


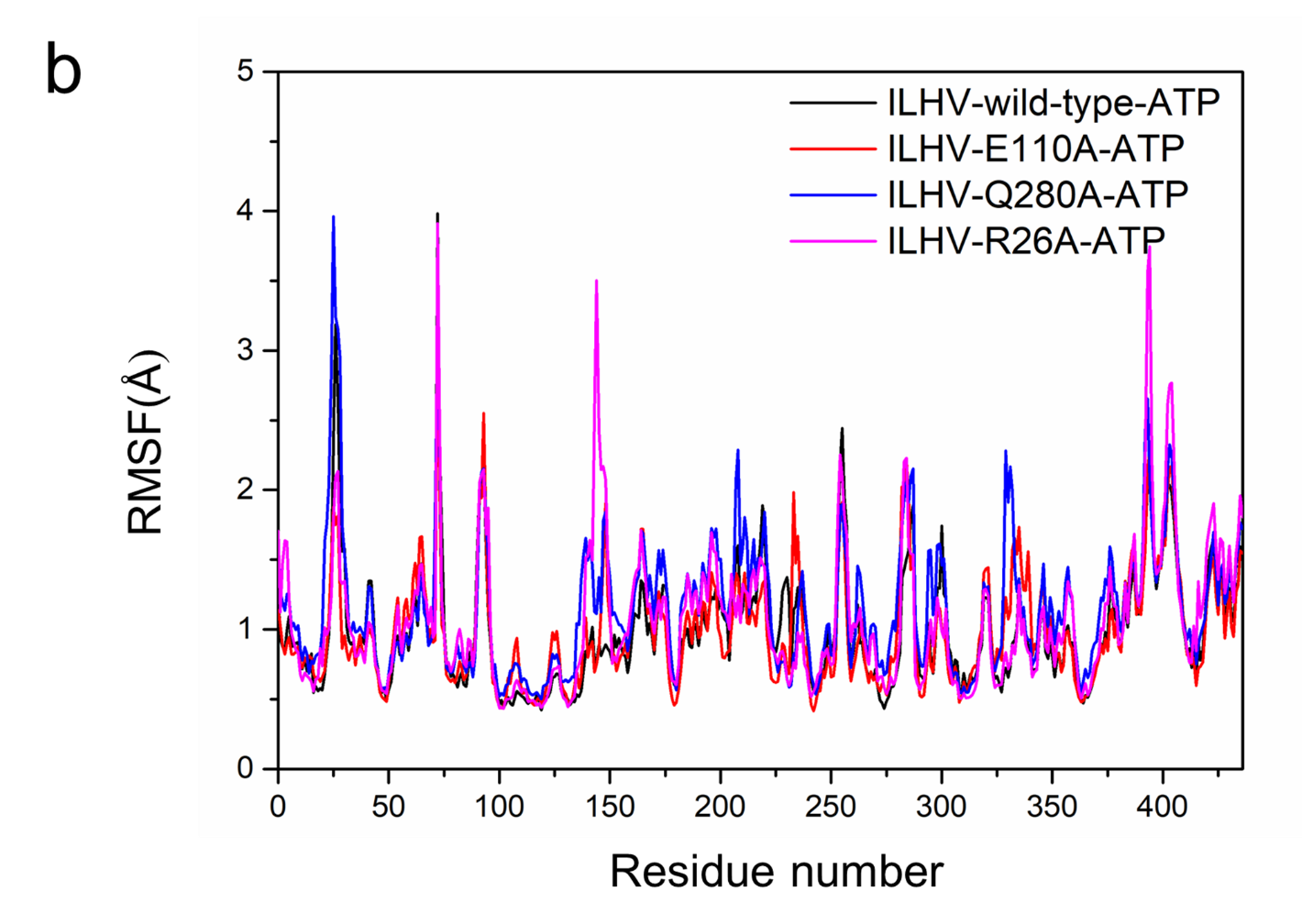


b

**Figure S7. a**, RMSD plot during molecular dynamics simulations of protein with ATP. **b**, RMSF plot during molecular dynamics simulations of protein with ATP.


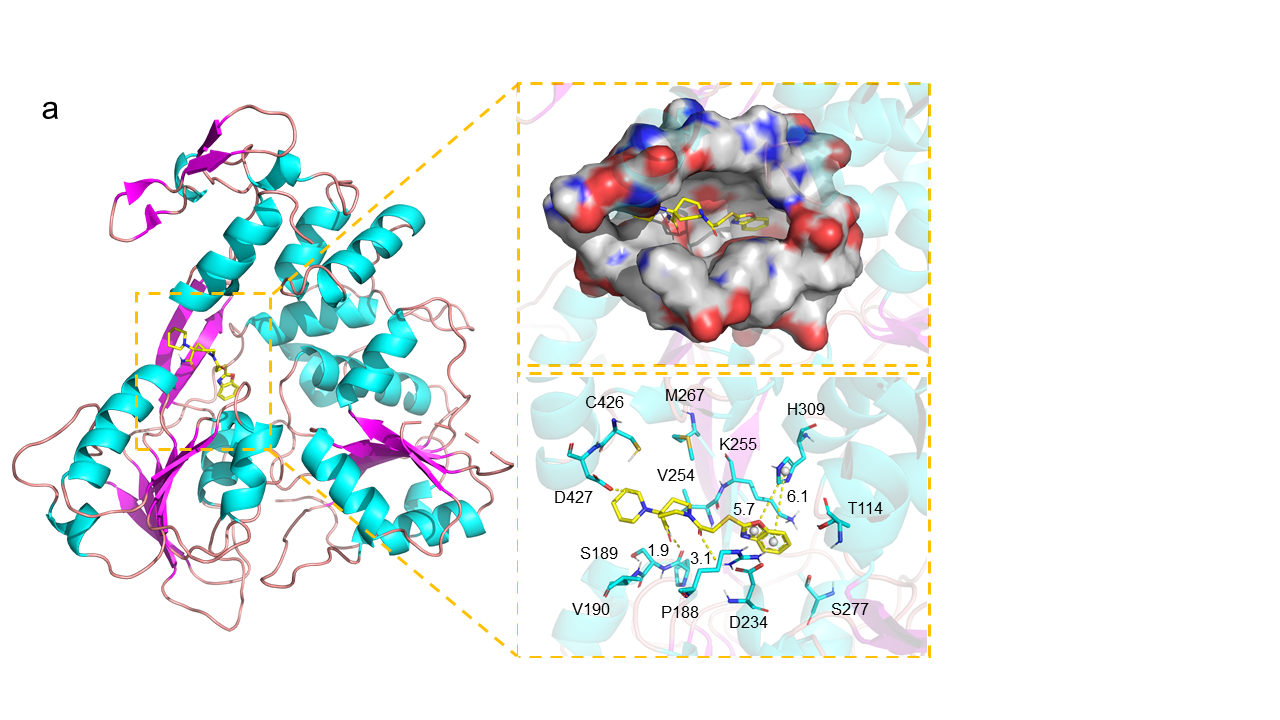


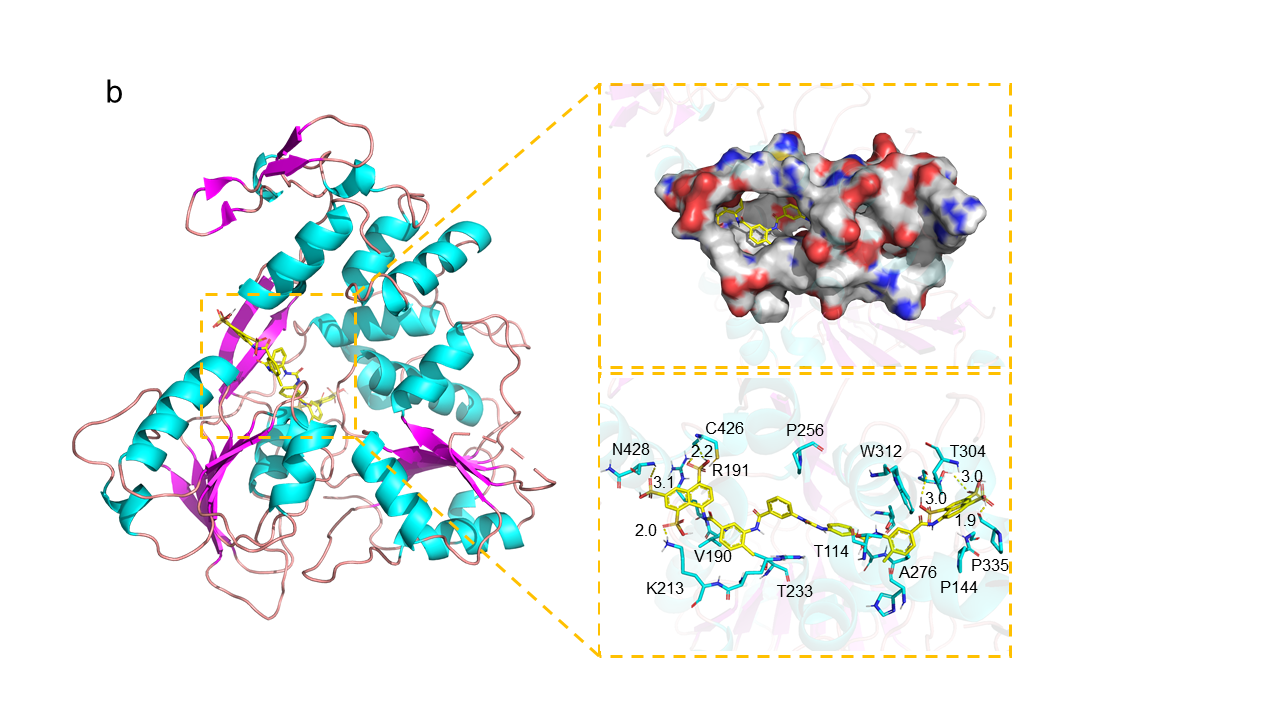


**Figure S8. a,** The binding mode of ILHV helicase with ST-610. **b,** The binding mode of ILHV helicase with Suramin.
